# Takotsubo cardiomyopathy complicated with apical thrombus formation on first day of the illness: a case report and literature review

**DOI:** 10.1186/s12872-017-0616-0

**Published:** 2017-07-03

**Authors:** H. M. M. T. B. Herath, S. P. Pahalagamage, Laura C. Lindsay, S. Vinothan, Sampath Withanawasam, Vajira Senarathne, Milinda Withana

**Affiliations:** 1 National Hospital, Colombo, Sri Lanka; 2 University of Edinburg, National Hospital, University of Edinburg, Scotland, Sri Lanka

**Keywords:** Takotsubo cardiomyopathy, Early left ventricular thrombus, Streptokinase, Case report

## Abstract

**Background:**

Takotsubo cardiomyopathy is characterized by transient systolic dysfunction of the apical and mid segments of the left ventricle in the absence of obstructive coronary artery disease. Intraventricular thrombus formation is a rare complication of Takotsubo cardiomyopathy and current data almost exclusively consists of isolated case reports and a few case series. Here we describe a case of Takotsubo cardiomyopathy with formation of an apical thrombus within 24 h of symptom onset, which has been reported in the literature only once previously, to the best of our knowledge. We have reviewed the available literature that may aid clinicians in their approach to the condition, since no published guidelines are available.

**Case presentation:**

A 68-year-old Sri Lankan female presented to a local hospital with chest pain. Electrocardiogram (ECG) showed ST elevation, and antiplatelets, intravenous streptokinase and a high dose statin were administered. Despite this ST elevation persisted; however the coronary angiogram was negative for obstructive coronary artery disease. Echocardiogram revealed hypokinesia of the mid and apical segments of the left ventricle with typical apical ballooning and a sizable apical thrombus. She had recently had a viral infection and was also emotionally distressed as her sister was recently diagnosed with a terminal cancer. A diagnosis of Takotsubo cardiomyopathy was made and anticoagulation was started with heparin and warfarin. The follow up echocardiogram performed 1 week later revealed a small persistent thrombus, which had completely resolved at 3 weeks.

**Conclusion:**

Though severe systolic dysfunction is observed in almost all the patients with Takotsubo cardiomyopathy, intraventricular thrombus formation on the first day of the illness is rare. The possibility of underdiagnosis of thrombus can be prevented by early echocardiogram in Takotsubo cardiomyopathy. The majority of reports found in the literature review were of cases that had formed an intraventriclar thrombus within the first 2 weeks, emphasizing the importance of follow up echocardiography at least 2 weeks later. The management of a left ventricular thrombus in Takotsubo cardiomyopathy is controversial and in most cases warfarin and heparin were used for a short duration.

**Electronic supplementary material:**

The online version of this article (doi:10.1186/s12872-017-0616-0) contains supplementary material, which is available to authorized users.

## Background

Takotsubo cardiomyopathy (TCM) or stress-induced cardiomyopathy is characterized by transient systolic dysfunction of the apical and mid segments of the left ventricle in the absence of obstructive coronary artery disease. In the typical type of stress-induced cardiomyopathy, contractility of the mid and apical segments of the left ventricle is depressed and there is balloon-like appearance of the distal ventricle with systole. TCM is much more common in postmenopausal women and is frequently triggered by unexpected emotional or physical stress [1]. Exaggerated sympathetic stimulation [2], catecholamine excess [3], coronary artery spasm and micro vascular dysfunction have been postulated as possible mechanisms in Takotsubo cardiomyopathy. It is a transient disorder and resolves with conservative treatment and supportive therapy. Intraventricular thrombus formation is a rare complication of Takotsubo cardiomyopathy and current data almost exclusively consist of isolated case reports and a few case series. In one case series, only 5 out of 95 patients (5.3%) with TCM developed left ventricular apical thrombus [4].

Here we describe a case of a postmenopausal female who presented with TCM who was initially treated as acute ST segment elevation myocardial infarction (STEMI). Despite administration of streptokinase and enoxaparin, a large left ventricular clot was found on the transthoracic echocardiogram (TTE) performed within 24 h. Rapid development of an apical thrombus within 24 h in TCM is rarely reported in literature and here we illustrate the importance of follow up TTE in these patients to recognize this complication. We also focus on the case reports of TCM complicated with intraventricular thrombus formation with regards to management, since no treatment guidelines are available and only the previous case reports and case series can be used to guide management.

## Case presentation

**Fig. 1:**
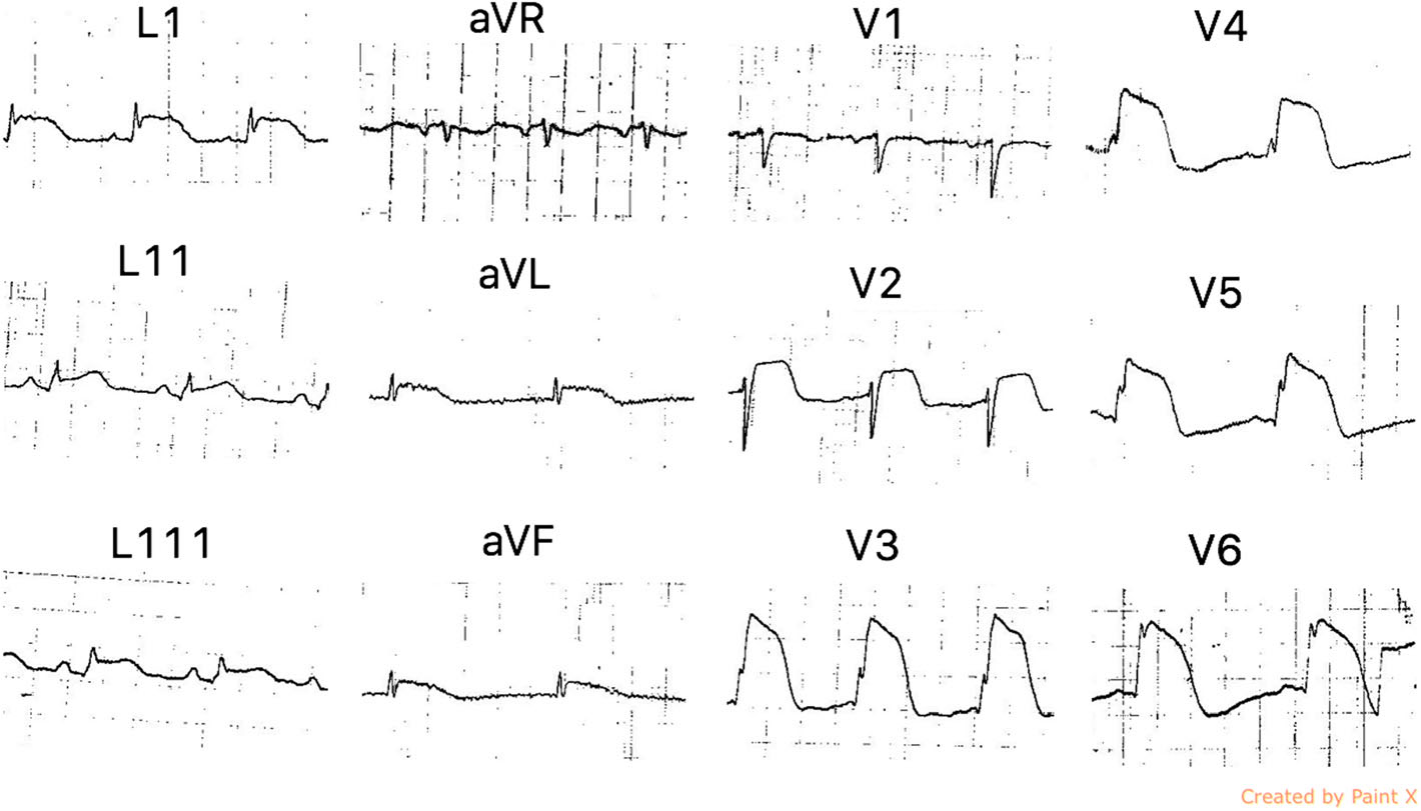
Electrocardiogram showing ST segment elevation in leads L1, aVL, L11, L111, aVF and V2 to V6

A 68-year-old Sri Lankan female (BMI 24.3 kg/ m^2^) was admitted to a local hospital, complaining of sudden onset severe retrosternal chest pain with autonomic symptoms. Electrocardiogram (ECG) showed ST segment elevations in leads LI, aVL, LII, LIII, aVF and V2 to V6 (Fig. 1). A diagnosis of acute STEMI was made and oral Aspirin 300 mg, Clopidogrel 300 mg, Atorvastatin 40 mg and intravenous streptokinase 1.5 MU were administered 2 h after the onset of chest pain followed by subcutaneous enoxaparin. The patient’s chest pain resolved, but due to persistent ST elevation on ECG, the patient was transferred to our cardiology unit for further management. She had been diagnosed with hypertension for 6 months and routine TTE performed 3 months previously showed normal ejection fraction and contractility. One week before this episode, she had 2 days of fever that was managed as viral illness at a local hospital. She was also emotionally stressed because her sister was recently diagnosed with a terminal cancer. She did not have any past history or family history of thromboembolic diseases or risk factors other than hypertension.

**Fig. 2:**
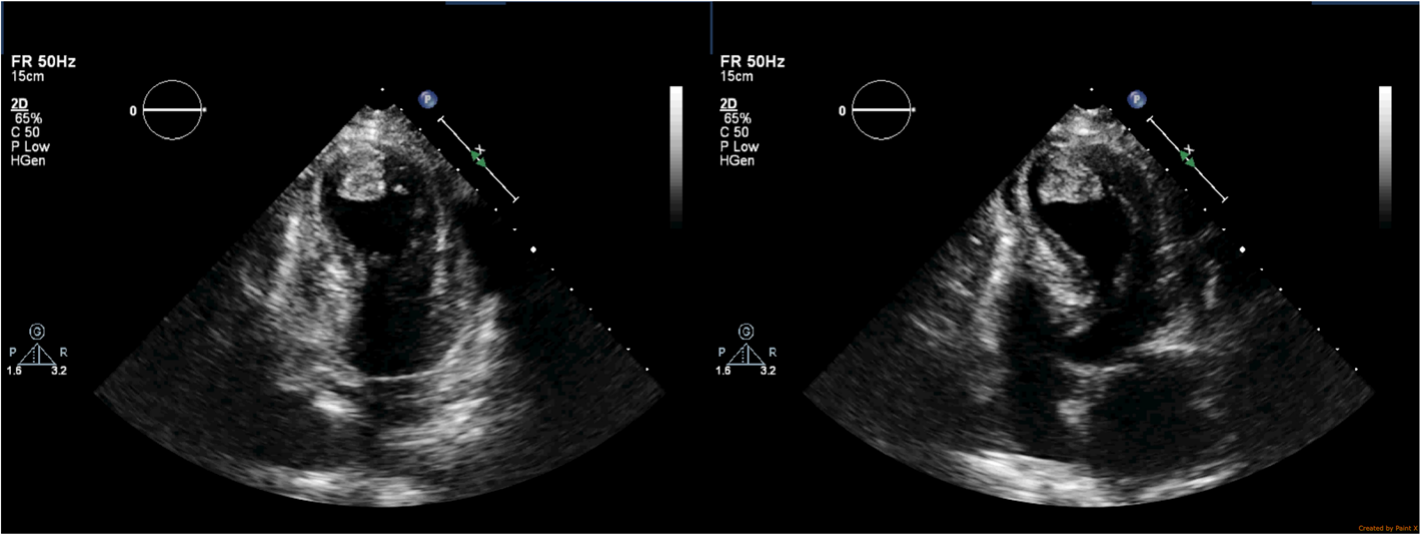
Transthoracic echocardiogram, performed 18 h after the onset of chest pain, showing hypokinesia of the *left* ventricular apex with apical ballooning. Ejection fraction was 40% and a 2.5 cm × 2 cm apical thrombus was detected

**Fig. 3:**
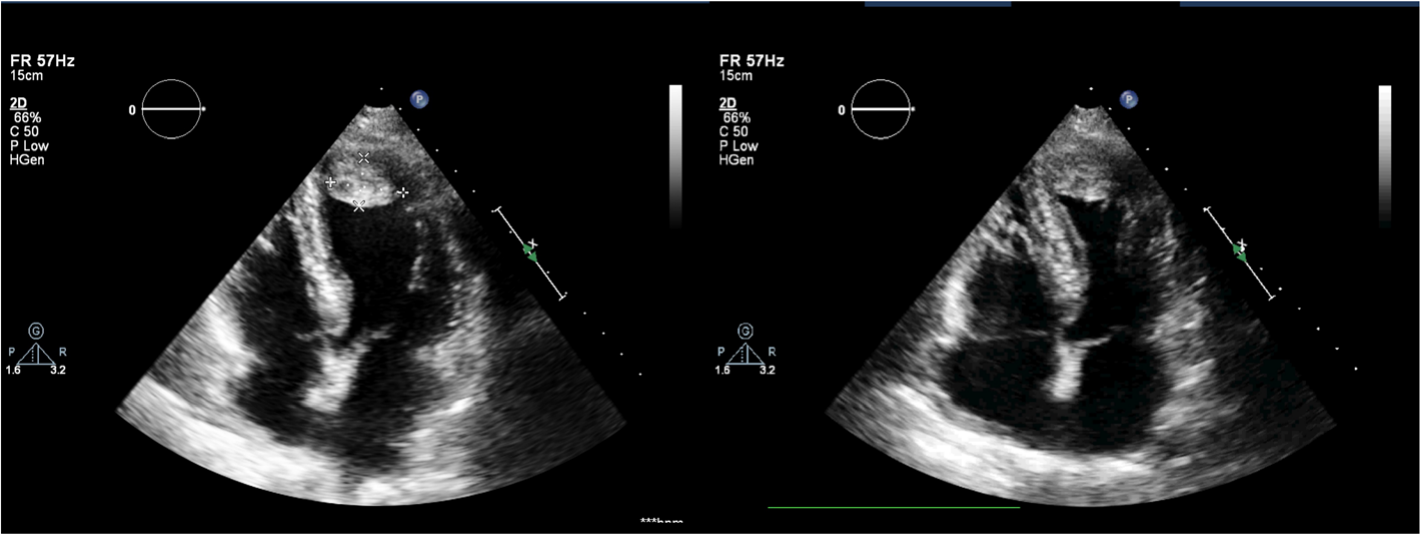
Follow up transthoracic echocardiogram, done 1 week later, showing only mild hypokinesia of the apex of the *left* ventricle and the thrombus which had reduced in size (2.1 cm × 1.8 cm)

**Fig. 4:**
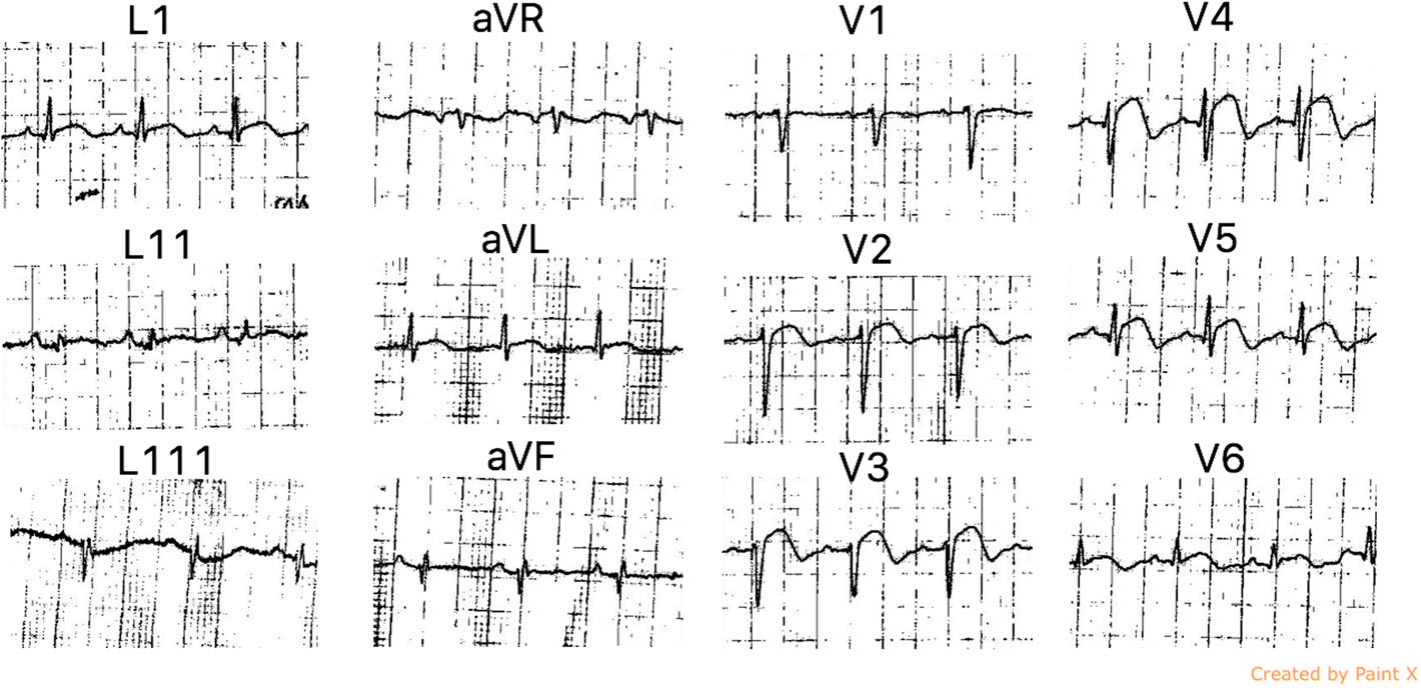
ECG done 1 week later showing resolving ST elevations



On examination, she was dyspnoeic at rest but maintained a saturation of more than 94% without supplemental oxygen. Her heart rate was 110 beats per minute and blood pressure was 100/60 mmHg. Jugular venous pressure was elevated and there were fine bibasal crepitations. TTE was performed 18 h after the onset of chest pain, which revealed hypokinesia of the mid and apical segments of the left ventricle with typical LV apical ballooning. Ejection fraction was 40% and a 2.5 cm × 2 cm apical thrombus was detected (Fig. 2)(Additional file 1: Movie S1 and Additional file 2: Movie S2). Because the coronary angiogram performed at the same time showed normal coronary arteries, Takotsubo cardiomyopathy was diagnosed and she was commenced on metoprolol, losartan, atorvastatin, diuretics and aspirin. We continued subcutaneous enoxaparin 1 mg/ kg bd and added warfarin 5 mg daily. Her full blood count (hemoglobin 12 g/dL, white blood cell count 7.79 × 10^9/^ L, platelet count 222 × 10^9/^ L), renal function tests, liver function tests, thyroid function tests, serum calcium, serum magnesium and coagulation profile were within normal range. The greatest troponin I value was 2.2 ng/ml (normal <0.5 ng/ml). Erythrocyte sedimentation rate was 13 mm in the first hour and C-reactive protein (CRP) was 6.7 mg/L (normal <5 mg/L). Human immunodeficiency virus serology, venereal disease research laboratory and hepatitis serology were also negative. After achieving a therapeutic international normalized ratio of 2–3, enoxaparin was omitted. Her symptoms improved gradually over 1 week. Follow up TTE performed 1 week later showed only mild hypokinesia of the apex of the left ventricle and the thrombus had reduced in size (2.1 cm × 1.8 cm) (Fig. 3). ECG also showed resolving ST elevation (Fig. 4). After 3 weeks, TTE showed normally contracting ventricles and the thrombus had resolved. We discontinued warfarin and continued with the other drugs. She did not experience any thromboembolism.

**Additional file 1: Movie S1.** TTE performed 18 h after the onset of chest pain, revealing hypokinesia of the mid and apical segments of the left ventricle with typical LV apical ballooning. Ejection fraction was 40% and a 2.5 cm × 2 cm apical thrombus was detected. (AVI 17890 kb)

**Additional file 2: Movie S2.** TTE performed 18 h after the onset of chest pain, revealing hypokinesia of the mid and apical segments of the left ventricle with typical LV apical ballooning. Ejection fraction was 40% and a 2.5 cm × 2 cm apical thrombus was detected. (AVI 15305 kb)


## Discussion

TCM was diagnosed in this patient who had clinical manifestations and ECG abnormalities out of proportion to the cardiac biomarkers with typical apical ballooning evident in TTE and normal coronary angiography [5]. We assumed this event was precipitated by emotional stress due to social problems and the recent upper respiratory tract infection. We assumed that the ventricular thrombus developed due to apical hypokinesia since the TTE performed 3 months earlier was normal and the thrombus was visualized at the apex as in other cases of TCM. The thrombus also resolved rapidly indicating that it was a newly formed thrombus. The other possible mechanism for thrombus formation in this patient is reduced wall motion due to myocarditis following viral flu she had 1 week back. But this less likely since she did not have symptoms, signs or ECG changes suggestive of cardiac involvement during that admission.

Apical thrombosis complicating TCM was first described in 2003 [6, 7]. Several isolated case reports and 2 case series were published later [4, 8]. A systematic review done in 2008 analyzed 15 patients with left ventricular thrombus formation in TCM. In all 15 cases thrombus was located in the left ventricular apical region and complete thrombus resolution was documented in every patient [9]. Here we summarise 50 cases of takotsubo cardiomyopathy complicated with ventricular thrombosis reported in the literature from 2003 to 2017 (Table 1). Like our patient, the majority of the cases was female (45 out of 49 patients; 92%) and was above 60 years of age (30 out of 49; 61%).

**Table 1:** Case report review of takotsubo cardiomyopathy complicated with ventricular thrombus formation from 2003 to 2017

Year	Age (Years)	Gender	Day of diagnosis^a^	Site of the thrombus	Treatment^b^	Time for resolution^c^	Ejection Fraction %	Thrombo Embolism^d^	Reference
2003	74	F	4	4 × 4 mm, LV apex	Warfarin	2w		Right hemiparesis	[6]
2003	64	F	2	LV	Anticoagulation	12w	40		[7]
2004	76	F	6		Heparin and warfarin	2w			[35]
2004	57	F	2	LV apex, 2.0 × 1.5 cm	Heparin and warfarin	4w		Right upper limb hemiplegia	[36]
2004	44	F	11 weeks	LV apex	Urokinase, warfarin			Renal infarct	[17]
2006	64	F		LV apex, 13 × 8 mm	Anticoagulant	4w			[37]
2006	76	F		LV apex	Anticoagulant	3w	35		[38]
2007	54	F	2	LV apex	Heparin and warfarin	1w			[15]
2007	74	F	3	LV apex	Heparin and warfarin	12d	33		[39]
2007	70	F	3	LV apex	Anticoagulant	3 m		Sensory aphasia	[40]
2007	74	M	14	20 × 15 mm LV apex	Anticoagulant	7w	30		[10]
2007	74	F		LV apex	Anticoagulant	2 m	40		[41]
2008	69	F	1	LV apex, Two mobile thrombi 5 × 6 mm and 8 × 10 mm	Heparin and phenprocoumon	4w	39		[8]
2008	69	F	8	Mobile thrombus adjacent to the posteromedial papillary muscle	Heparin	9d	30		[8]
2008	43	F	4	LV apex	Heparin	11d	34		[8]
2008	69	F	3	28 × 22 mm anterior and anteroseptal wall, 4 × 4 mm mobile thrombus adjacent to the anterolateral papillary muscle	Heparin	27d	45		[8]
2008	55	F	2	LV apex	Heparin and warfarin	1 m			[19]
2008	53	F		LV apex	Heparin aspirin	2w	32		[42]
2008	74	F		Multiple thrombotic masses	Warfarin	2w	35	Dysphasia, right arm paresis	[26]
2008	43	F	5	LV apex	Heparin and warfarin	8d	<25	Right renal infarct	[43]
2008	64	F		LV apex	Warfarin		45	Broca’s aphasia	[44]
2009	28	F	3	LV apex	Heparin and warfarin	3w	25		[20]
2009	57	M		LV apex	Heparin and warfarin	2w	48		[45]
2011	78 87 71 82 55	F F F F F		Mural in 2 cases and protruding in 3 cases	Anticoagulant	4w	45 ± 6%	Cerebral infarction in one patient	[4]
2011	62	F	35	LV apex	Heparin and warfarin	3 m	18		[16]
2011	76	F	8	LV apex, 24 × 25 mm	Heparin and warfarin	1w	18	Multiple brain embolic infarctions	[46]
2011	68	F	3	LV apex, 26 × 29 mm	Heparin and warfarin	12d			[47]
2011	69	F	6	LV apex	Anticoagulant	14d			[48]
2012	78	F		LV apex	Anticoagulant	2w	50		[49]
2012	70	F	13	LV apex	Anticoagulant		55		[50]
2012	29	F	2	Mid right ventricular cavity, 16 × 9 8 mm,	Anticoagulant	7d			[22]
2012	48	M	15	Apical inferior wall, 28 × 16 mm	Sx				[25]
2012	78	F	7	LV apex	Heparin	17d			[30]
2013	52	F	3	LV apex, 37 × 21 mm	Heparin and warfarin			Multifocal micro infarctions in the brain, pleen and kidneys	[27]
2013	78	F		Attached to septoapical wall, 30 × 15 mm	Aspirin, heparin		35	Left hemiparesis and dysarthria, large thrombus at the trunk and branches of the superior mesenteric artery	[24]
2013	50	M		LV apex, 3.6 cm × 1.7 cm	Enoxaparin, Clopidogrel and warfarin	7w	45	Dense left sided hemiplegia, left homonymous hemianopia, aphasia	[31]
2013	63	F	3 weeks	LV apex, 1.10 × 2.12 cm	Heparin and warfarin	3 m	43		[18]
2013	58	F	2		Heparin, warfarin and sx				[33]
2015	66	F		LV apex	Heparin, acetyl-salicylic acid and clopidogrel	17d		Ischemic infarctions of the left median cerebral artery	[51]
2015	59	F	13	LV apex	Heparin, Warfarin, sx		<25		[21]
2016	48	F		LV apex	Anticoagulant	3 m		Right femoral artery embolism	[52]
2016	57	F	4	LV apex, 2.3 × 3.3 cm	Warfarin	15d	35		[53]
2016	61	F		LV apex	Heparin and warfarin		35		[14]
2016	55	F		LV apex, 20 × 10 mm	Warfarin	3 m			[54]
2017	48	F		LV apex	Heparin and warfarin		30		[55]
2017	88	F		Biventricular	Heparin		35		[21]
2003	74	F	4	4 × 4 mm, LV apex	Warfarin	2w		Right hemiparesis	[6]
2003	64	F	2	LV	Anticoagulation	12w	40		[7]
2004	76	F	6		Heparin and warfarin	2w			[35]
2004	57	F	2	LV apex, 2.0 × 1.5 cm	Heparin and warfarin	4w		Right upper limb hemiplegia	[36]
2004	44	F	11 weeks	LV apex	Urokinase, warfarin			Renal infarct	[17]
2006	64	F		LV apex, 13 × 8 mm	Anticoagulant	4w			[37]
2006	76	F		LV apex	Anticoagulant	3w	35		[38]
2007	54	F	2	LV apex	Heparin and warfarin	1w			[15]
2007	74	F	3	LV apex	Heparin and warfarin	12d	33		[39]
2007	70	F	3	LV apex	Anticoagulant	3 m		Sensory aphasia	[40]
2007	74	M	14	20 × 15 mm LV apex	Anticoagulant	7w	30		[10]
2007	74	F		LV apex	Anticoagulant	2 m	40		[41]
2008	69	F	1	LV apex, Two mobile thrombi 5 × 6 mm and 8 × 10 mm	Heparin and phenprocoumon	4w	39		[8]
2008	69	F	8	Mobile thrombus adjacent to the posteromedial papillary muscle	Heparin	9d	30		[8]
2008	43	F	4	LV apex	Heparin	11d	34		[8]
2008	69	F	3	28 × 22 mm anterior and anteroseptal wall, 4 × 4 mm mobile thrombus adjacent to the anterolateral papillary muscle	Heparin	27d	45		[8]
2008	55	F	2	LV apex	Heparin and warfarin	1 m			[19]
2008	53	F		LV apex	Heparin aspirin	2w	32		[42]
2008	74	F		Multiple thrombotic masses	Warfarin	2w	35	Dysphasia, right arm paresis	[26]
2008	43	F	5	LV apex	Heparin and warfarin	8d	<25	Right renal infarct	[43]
2008	64	F		LV apex	Warfarin		45	Broca’s aphasia	[44]
2009	28	F	3	LV apex	Heparin and warfarin	3w	25		[20]
2009	57	M		LV apex	Heparin and warfarin	2w	48		[45]
2011	78 87 71 82 55	F F F F F		Mural in 2 cases and protruding in 3 cases	Anticoagulant	4w	45 ± 6%	Cerebral infarction in one patient	[4]
2011	62	F	35	LV apex	Heparin and warfarin	3 m	18		[16]
2011	76	F	8	LV apex, 24 × 25 mm	Heparin and warfarin	1w	18	Multiple brain embolic infarctions	[46]
2011	68	F	3	LV apex, 26 × 29 mm	Heparin and warfarin	12d			[47]
2011	69	F	6	LV apex	Anticoagulant	14d			[48]
2012	78	F		LV apex	Anticoagulant	2w	50		[49]
2012	70	F	13	LV apex	Anticoagulant		55		[50]
2012	29	F	2	Mid right ventricular cavity, 16 × 9 8 mm,	Anticoagulant	7d			[22]
2012	48	M	15	Apical inferior wall, 28 × 16 mm	Sx				[25]
2012	78	F	7	LV apex	Heparin	17d			[30]
2013	52	F	3	LV apex, 37 × 21 mm	Heparin and warfarin			Multifocal micro infarctions in the brain, spleen and kidneys	[27]
2013	78	F		Attached to septoapical wall, 30 × 15 mm	Aspirin, heparin		35	Left hemiparesis and dysarthria, large thrombus at the trunk and branches of the superior mesenteric artery	[24]
2013	50	M		LV apex, 3.6 cm × 1.7 cm	Enoxaparin, Clopidogrel and warfarin	7w	45	Dense left sided hemiplegia, left homonymous hemianopia, aphasia	[31]
2013	63	F	3 weeks	LV apex, 1.10 × 2.12 cm	Heparin and warfarin	3 m	43		[18]
2013	58	F	2		Heparin, warfarin and sx				[33]
2015	66	F		LV apex	Heparin, acetyl-salicylic acid and clopidogrel	17d		Ischemic infarctions of the left median cerebral artery	[51]
2015	59	F	13	LV apex	Heparin, Warfarin, sx		<25		[21]
2016	48	F		LV apex	Anticoagulant	3 m		Right femoral artery embolism	[52]
2016	57	F	4	LV apex, 2.3 × 3.3 cm	Warfarin	15d	35		[53]
2016	61	F		LV apex	Heparin and warfarin		35		[14]
2016	55	F		LV apex, 20 × 10 mm	Warfarin	3 m			[54]
2017	48	F		LV apex	Heparin and warfarin		30		[55]
2017	88	F		Biventricular	Heparin		35		[21]
2003	74	F	4	4 × 4 mm, LV apex	Warfarin	2w		Right hemiparesis	[6]
2003	64	F	2	LV	Anticoagulation	12w	40		[7]
2004	76	F	6		Heparin and warfarin	2w			[34]
2004	57	F	2	LV apex, 2.0 × 1.5 cm	Heparin and warfarin	4w		Right upper limb hemiplegia	[35]
2004	44	F	11 weeks	LV apex	Urokinase, warfarin			Renal infarct	[17]
2006	64	F		LV apex, 13 × 8 mm	Anticoagulant	4w			[36]
2006	76	F		LV apex	Anticoagulant	3w	35		[37]
2007	54	F	2	LV apex	Heparin and warfarin	1w			[15]
2007	74	F	3	LV apex	Heparin and warfarin	12d	33		[38]
2007	70	F	3	LV apex	Anticoagulant	3 m		Sensory aphasia	[39]
2007	74	M	14	20 × 15 mm LV apex	Anticoagulant	7w	30		[10]
2007	74	F		LV apex	Anticoagulant	2 m	40		[40]
2008	69	F	1	LV apex, Two mobile thrombi 5 × 6 mm and 8 × 10 mm	Heparin and phenprocoumon	4w	39		[8]
2008	69	F	8	Mobile thrombus adjacent to the posteromedial papillary muscle	Heparin	9d	30		[8]
2008	43	F	4	LV apex	Heparin	11d	34		[8]
2008	69	F	3	28 × 22 mm anterior and anteroseptal wall, 4 × 4 mm mobile thrombus adjacent to the anterolateral papillary muscle	Heparin	27d	45		[8]
2008	55	F	2	LV apex	Heparin and warfarin	1 m			[19]
2008	53	F		LV apex	Heparin aspirin	2w	32		[41]
2008	74	F		Multiple thrombotic masses	Warfarin	2w	35	Dysphasia, right arm paresis	[24]
2008	43	F	5	LV apex	Heparin and warfarin	8d	<25	Right renal infarct	[42]
2008	64	F		LV apex	Warfarin		45	Broca’s aphasia	[43]
2009	28	F	3	LV apex	Heparin and warfarin	3w	25		[44]
2009	57	M		LV apex	Heparin and warfarin	2w	48		[45]
2011	78 87 71 82 55	F F F F F		Mural in 2 cases and protruding in 3 cases	Anticoagulant	4w	45 ± 6%	Cerebral infarction in one patient	[4]
2011	62	F	35	LV apex	Heparin and warfarin	3 m	18		[16]
2011	76	F	8	LV apex, 24 × 25 mm	Heparin and warfarin	1w	18	Multiple brain embolic infarctions	[46]
2011	68	F	3	LV apex, 26 × 29 mm	Heparin and warfarin	12d			[47]
2011	69	F	6	LV apex	Anticoagulant	14d			[48]
2012	78	F		LV apex	Anticoagulant	2w	50		[49]
2012	70	F	13	LV apex	Anticoagulant		55		[50]
2012	29	F	2	Mid right ventricular cavity, 16 × 9 8 mm,	Anticoagulant	7d			[20]
2012	48	M	15	Apical inferior wall, 28 × 16 mm	Sx				[23]
2012	78	F	7	LV apex	Heparin	17d			[28]
2013	52	F	3	LV apex, 37 × 21 mm	Heparin and warfarin			Multifocal micro infarctions in the brain, spleen and kidneys	[25]
2013	78	F		Attached to septoapical wall, 30 × 15 mm	Aspirin, heparin		35	Left hemiparesis and dysarthria, large thrombus at the trunk and branches of the superior mesenteric artery	[22]
2013	50	M		LV apex, 3.6 cm × 1.7 cm	Enoxaparin, Clopidogrel and warfarin	7w	45	Dense left sided hemiplegia, left homonymous hemianopia, aphasia	[29]
2013	63	F	3 weeks	LV apex, 1.10 × 2.12 cm	Heparin and warfarin	3 m	43		[18]
2013	58	F	2		Heparin, warfarin and sx				[31]
2015	66	F		LV apex	Heparin, acetyl-salicylic acid and clopidogrel	17d		Ischemic infarctions of the left median cerebral artery	[51]
2015	59	F	13	LV apex	Heparin, Warfarin, sx		<25		[32]
2016	48	F		LV apex	Anticoagulant	3 m		Right femoral artery embolism	[52]
2016	57	F	4	LV apex, 2.3 × 3.3 cm	Warfarin	15d	35		[53]
2016	61	F		LV apex	Heparin and warfarin		35		[14]
2016	55	F		LV apex, 20 × 10 mm	Warfarin	3 m			[54]
2017	48	F		LV apex	Heparin and warfarin		30		[55]
2017	88	F		Biventricular	Heparin		35		[21]

*F* female, *M* male,

Day of diagnosis^a^ = the date of diagnosis from the onset of symptoms / diagnosis of takotsubo cardiomyopathy, given in number of days, in 2 cases given in weeks

Treatment^b^ = anticoagulant = In case reports which has not specified the anticoagulant used, Sx = ventriculotomy and surgical thrombectomy

Time for resolution^c^ = *d* days, *w* weeks, *m* months

Thrombo Embolism^d^ = Thrombo embolic episodes diagnosed after the detection of ventricular thrombus

Abnormality in the contraction of the left ventricular apical region resulting in transient apical aneurysm and local hemostasis [4], endocardial injury with local exposure or release of thrombogenic substances [10] and influence of catecholamines on nucleotide-induced platelet aggregation [11, 12] have been postulated as possible mechanisms for the thrombus formation. In TCM, ventricular apical aneurysm always occurs during the acute phase and is often more extensive than in acute myocardial infarction. Plasma catecholamine levels are also much higher in the TCM than in acute coronary syndrome. These might be the causes why out patient developed apical thrombus in the very acute phase.

In our patient, the thrombus was detected using TTE. Ventriculography or cardiac magnetic resonance imaging can also be used to recognize this complication. The cardiac magnetic resonance imaging (MRI) features of a thrombus in TCM was first described by Singh, V. et al. [10]. Cardiac MRI [13] and contrast CT [14] have been used to identify ventricular thrombi that are not visualized by echocardiography and provide more information on the myocardium. In our patient, we did not perform cardiac MRI or CT and ventriculography was not performed due to increased risk of thromboembolism.

The significant feature in our case is the rapid development of ventricular thrombus within 24 h, despite administration of streptokinase and heparin. We could only find one other reported case, which described a ventricular thrombus found on TTE performed within 24 h from the onset of symptoms [8]. Kimura, K. et al., had reported a giant apical thrombus which had formed within 2 days [15]. In all but 3 cases the thrombus was identified within 14 days. Lee, P.H. et al., had reported a case of TCM in which a newly developed apical thrombus was noted 5 weeks later in serial TTE which is the longest time period reported in literature [16]. This patient had a multi-septated liver abscess with adjacent hepatic venous thrombosis, a very low ejection fraction of 18% and had to be treated at the medical intensive care unit with inotropic support. Another case report describes a patient who developed a renal infarct 11 weeks after TCM, with TTE demonstrating a thrombus attached to the left ventricular apical wall [17]. Here serial TTE had not been performed, so the exact time taken for the development of thrombus was not certain. This patient had a bicuspid aortic valve and aortic regurgitation, so the author highlights that a ventricular thrombus should be considered not only as an early but also as a delayed complication of TCM, especially in a patient with organic heart disease. Shim, I.K. et al., reported a case in which an apical thrombus was visualized on TTE performed 3 weeks after TCM [18]. This patient had a 25-year history of systemic lupus erythematosus and the apical ballooning persisted for more than 3 weeks.

We were unable to find any case reports of TCM complicated with thrombus formation despite administration of streptokinase and heparin on admission. In one case report, TTE performed 24 h later revealed a solid thrombus in the akinetic apical region of the left ventricle [19] despite an oral dose of aspirin 300 mg and a bolus of intravenous heparin 4000 U given on admission. In another case report, an apical clot was visualized on day 3 and in this patient, aspirin, intravenous heparin, and glycoprotein IIb/IIIa inhibitor was started on admission, but stopped on the same day [20]. Niino, T. et al., reported a case in which TTE revealed an apical thrombus on day 13 and this patient received heparin from day 1 to day 6 [21]. One patient had developed a thrombus while on full dose of low molecular weight heparin [8].

As in most cases described in literature, in our patient a single thrombus was visualized at the apex of the left ventricle. Only one case report described a thrombus in the right ventricular cavity attached to the akinetic right ventricular free wall [22]. A recent case repot describes a 88 years old female with biventricular TCM complicated by biventricular thrombosis [23]. A thrombus attached to the septo-apical wall [24], a thrombus attached to the apical inferior wall by a thin stalk [25], a thrombus attached to an akinetic segment of the anterior and anteroseptal wall, a mobile thrombus adjacent to the anterolateral papillary muscle [8], a mobile thrombus adjacent to the posteromedial papillary muscle [8], two mobile thrombi in the left ventricular apex [8] and multiple thrombotic masses in the left ventricular apex [26] were also described.

The feared complication of a left ventricular thrombus is embolisation and fortunately our patient did not have any embolic events, which was probably prevented by early treatment. Out of the 49 cases we summarized, 8 cases had isolated cerebral thromboemboli, one case had an isolated renal infarct [17], one case had multifocal micro infarctions in the brain, spleen and kidneys [27] and one case had cerebral and superior mesenteric artery thromboembolism [24] (Table 1). The management of TCM with ventricular thrombus is directed to prevent embolic episodes and in most cases heparin and warfarin were used for anticoagulation (Table 1). In one case, urokinase was used for lysis of the thrombus [17]. Since no guidelines are available for management, indirect data can be used from randomized trials that evaluated anticoagulation to prevent left ventricular thrombus formation and embolisation in patients with acute myocardial infarction. For patients with anterior myocardial infarction and left ventricular thrombus or at high risk for left ventricular thrombus (ejection fraction less than 40%, antero- apical wall motion abnormality) American College of Chest Physician’s Evidence-Based Clinical Practice Guidelines recommend warfarin (plus antiplatelet for ischemic heart disease) [28]. The duration of warfarin therapy for these patients with acute myocardial infarction is at least for 3 months according to guidelines. However the wall motion abnormalities in TCM are known to improve rapidly and completely compared to acute myocardial infarction, so the optimum duration of anticoagulation in not clear - in most cases thrombus resolved within 1 month (39 out of 49; 80%) and in all cases the thrombus resolved within 3 months. Serial TTE was performed for the majority of cases to confirm thrombus resolution. We could find only one case report describing repeated embolic events despite anticoagulation with subcutaneous enoxaparin and aspirin treatment [24]. Myocardial necrosis and cardiac rupture [29], massive hemorrhagic effusion following ventricular wall rupture [30], large cerebral infarct with mass effect and hemorrhagic transformation [31] can complicate the medical management. Most thrombi described were smooth, conform to the cavity shape and are relatively stable. Thrombectomy is rarely recommended if they are mobile or pedunculated, due to the high risk of embolization [32]. Ventriculotomy and surgical thrombectomy was only indicated in 3 of the reported cases [21, 25, 33]. Based on the available evidence, we commenced enoxaparin with warfarin and the thrombus resolved in 3 weeks following which anticoagulation was omitted.

Use of prophylactic anticoagulation to prevent thrombus formation in TCM is not practiced and no specific clinical, radiological or biochemical marker is available to risk categorize these patients. Haghi, D. et al., have stated that elevated serum CRP levels and thrombocytosis indicate higher risk of developing thrombi [8] and Ouchi, K. et al., have suggested D-dimer levels as a screening test for thrombosis [14]. In our patient, CRP was not significantly elevated and the platelet count was normal. Only a few case reports are available, and in most of them full biochemical analysis was not performed, limiting our ability to formulate risk factors to predict thrombus formation in TCM. No particular features to predict the occurrence of left ventricular thrombosis were identified in the only published systematic review either [9]. The number of echocardiograms performed in a patient, the operator skill and the use of cardiac MRI and CT influence thrombus detection making the determination of the true incidence of left ventricular thrombosis in TCM difficult, again limiting recommendations regarding prophylactic anticoagulation. Since most of the patients with TCM present with chest pain and ST segment elevation [34], the chances of receiving thrombolytic therapy, antiplatelets and anticoagulation on presentation are high, as was the case for our patient. This may reduce the chance of thrombus formation, because the majority of those who developed the complication had not received any form of anticoagulation prior to detection of the thrombus.

## Conclusion

Although severe systolic dysfunction is observed in almost all patients with TCM, intraventricular thrombus formation is rarely reported in the literature. Most thrombi were detected during the first 2 weeks, emphasising the importance of follow up echocardiography at least 2 weeks later. The management of a left ventricular thrombus in TCM is controversial and in most cases warfarin and heparin is used for a short duration. Most of the thrombi resolved within 2 weeks of therapy and serial TTE can be used to monitor response. The role of prophylactic anticoagulants in TCM and risk factors to predict thrombosis should be examined further as current data is not enough to formulate a firm recommendation.

## Abbreviations

CRP: C-reactive protein
ECG: electrocardiogram
MRI: magnetic resonance imaging
STEMI: ST elevation myocardial infarction
TCM: Takotsubo cardiomyopathy
TTE: transthoracic echocardiogram
